# Analysis of factors related to browning of red sour soup during fermentation

**DOI:** 10.3389/fnut.2023.1092745

**Published:** 2023-02-28

**Authors:** Zhiqi Liu, Xiaojie Zhou, Ming Wen, Zhouliang Gong, Bilian Lin, Liangzhong Zhao, Jianrong Wang

**Affiliations:** ^1^College of Food and Chemical Engineering, Shaoyang University, Shaoyang, China; ^2^Hunan Provincial Key Laboratory of Soybean Products Processing and Safety Control, Shaoyang University, Shaoyang, China; ^3^Shenzhen Raink Ecology & Environment Co., Ltd., Shenzhen, China

**Keywords:** red sour soup, fermentation, non-enzymatic browning, polyphenol, path analysis

## Abstract

As a traditional fermentation food, red sour soup (RSS) is very popular in China. However, browning is always occurred during the process of fermentation, which influences the sensory quality of RSS and limits its further application. Thus, it is meaningful to elucidate the main factors related to browning during the process of fermentation. Herein, the changes in various factors related to browning from group spontaneous (RSS-SF) and inoculant fermentation (RSS-IF) were determined and analyzed. Firstly, the activity changes of enzymes related to browning indicated that browning of group RSS-SF and RSS-IF during fermentation was not related to enzymatic browning. Secondly, path analysis revealed that the main factors related to non-enzymatic browning of group RSS-SF and RSS-IF were oxidation of polyphenol and degradation of ascorbic acid (Vc). The results of this study not only identifies the main factors associate with browning of RSS, but also provides foundation on how to control the browning of RSS in further study.

## Introduction

As a traditional fermentation food of Miao and Dong nationalities, sour soup is very popular in China. Briefly, sour soup mainly includes white and red sour soup (1, 2). The raw materials for white sour soup (WSS) are mainly from rice slurry. Different from WSS, red sour soup (RSS) is usually made by fermenting red chili peppers and tomato (3, 4). Recently, a series of studies exhibited that RSS is abundant in bioactive substances such as polyphenol, minerals and vitamins (5). Thus, it is considered that RSS may have variety of bioactivities, including anti-oxidation, enhancement of immunity, anti-aging as well as maintenance of healthy intestinal microbiota (3, 4). So far, the methods for preparation of RSS are mainly composed of spontaneous and inoculant fermentation. No matter whether spontaneous or inoculant fermentation, browning is always occurred during the process of preparation of RSS. As an important indicator of RSS, color plays an important role on influencing its acceptability and desirability to consumers. Thus, it is necessary to elucidate the key factors related to browning during the process of fermentation.

In summary, there are two types of browning in fruits and vegetables, including enzymatic and non-enzymatic browning. According to previous literature, enzymatic browning is always involved in polyphenoloxidase (PPO) and phenolic compounds (6). As the vegetables and fruits are cut or peeled, the PPO is released. Then, the phenolic compounds are converted into brown pigments by PPO (7). Usually, enzymatic browning is readily occurred when the pH is in the range from pH 5.0 to 7.0. For comparison, non-enzymatic browning is more complicated than enzymatic browning. Generally, non-enzymatic browning is composed of Maillard reaction, caramel reaction, ascorbic acid (Vc) oxidation-decomposition, oxidation of lipid as well as polyphenol chemical oxidation (8). It was observed that the reason for non-enzymatic of different food varies greatly. For instance, the browning of orange juice is closely related to Maillard reaction during the process of storage (9). However, the main factors contributed to browning of Dongbei Suancai are degradation of ascorbic acid and oxidation-polymerization of polyphenols (10). Moreover, the oxidation of lipid could cause the non-enzymatic browning in various foods (11).

As the prevalence of RSS, it has received extensive and increasing attention. To date, the research related to RSS are mainly focused on microorganism communities (1, 12), and flavor (4). However, there are no reports concentrate on the changes of browning during the process of fermentation. Moreover, it is meaningful to understand the details of browning of RSS during the process of fermentation which is necessary for improving browning control. In this study, the key factors related to browning were determined during the process of fermentation and the main factors contributed to browning of RSS were isolated by path analysis method. The results of this study will elucidate the main factors associate with browning of RSS and provide foundation on how to control the browning of RSS during fermentation.

## Materials and methods

The *Lactobacillus rhamnosus* was isolated and identified by 16sRNA and conserved our laboratory. The raw materials for RSS, such as tomatoes, chili pepper, garlic and ginger, were purchased from supermarket (Shaoyang, China). The gallic acid, ascorbic acid, 2, 6-dichlorophenol indophenol, oxophenic acid, methyl catechol, L-phenylalanine, linoleic acid were purchased from Beijing Solarbio Co., Ltd. (Beijing, China). All the chemicals used in this study were of analysis grade.

### Preparation of red sour soup

The process for preparation of RSS was same as our previous study (13). For spontaneous fermentation (named RSS-SF), firstly, the tomatoes (3.5 kg) and chili peppers (0.5 kg) were washed, mixed and grinded into jam like liquid. Then, ingredients such as salt, wine, ginger, garlic, glucose and lactose were added into the obtained jam like liquid with ratio of 1, 1, 0.75, 0.75, 0.25, and 0.75% (w/v), respectively. Finally, the mixture was incubated at 85°C for 20 min and put into fermentation containers. The process of preparation of RSS for inoculant fermentation (named RSS-IF) was same as RSS-SF except addition with *L. rhamnosus*. The RSS-SF and RSS-IF both were fermented at 37°C for 28 days. Different samples were randomly withdrawn at 0, 3, 7, 14, 21, and 28 days for subsequent analysis.

### Determination of browning degree

The method for determination of browning degree was same as previous study (9, 10). To begin with, 10 g RSS was mixed with 20 mL 95% alcohol and homogenized for 20 min. After that, the mixture was centrifuged at 10,000 × g for 10 min at 4°C. Lastly, the obtained supernatant was monitored at 420 nm using a spectrophotometer (UV-2600, Shimadzu Company, Japan). The value of browning degree was according to equation 1.


(1) $$\mathrm{browning degree}=A_{420\mathrm{nm}}$$

where A_420nm_ is the absorbance at 420 nm.

### Determination of activity of enzymes related to browning

The activity of PPO (14), peroxidase (POD) (15), phenylalaninammo-nialyase (PAL) (16) and lipoxygenase (LOX) (16) were determined according to the already reported research and the detail processes for determination of those enzymes were provided in Supplementary material.

### Determination of amino nitrogen, carbonyl value, and ascorbic acid

The method for determination of amino nitrogen, carbonyl value and Vc were in accordance with previous study (10). The detail protocols for those methods were provided in Supplementary material.

### Determination of reducing sugar and total polyphenol

The methods for determination of reducing sugar (17) and total polyphenol (18) were based on the previous studies and the detail protocols for those methods were provided in Supplementary material.

### Statistical analysis

All results were presented as average values from three replications with ± standard deviation (SD). Data was analyzed by one way analysis of variance (ANOVA) of the SPSS 25.0 (International Business Machines Corporation, Amunk, New York, United States) and mean separations were performed by Duncan’s multiple-range test. Different letters were used to indicate the significant differences at *p* ≤ 0.05 of each group.

Path analysis was performed to investigate the main factors related to browning of RSS during fermentation. The content of amino nitrogen (*X*_1_), reducing sugar (*X*_2_), total polyphenol (*X*_3_), Vc (*X*_4_), carbonyl value (*X*_5_) were taken as the independent variables, and browning degree (*Y*) was taken as the dependent variable. The direct path coefficients (*P*_1_–*P*_5_) and indirect path coefficients (*P*_ij_) between all these independent variables and dependent variable were analyzed by SPSS 25.0 (International Business Machines Corporation., Amunk, New York, United States). The determination coefficients (*d*_ij_) reflected the degree of independent variables to dependent variable and were calculated by correlation coefficients and path coefficients. The determination coefficient indicated the influence degree of the factor on the result (expressed in d), and it can be determined by formula through correlation coefficient and path coefficient:


(2) $$\mathrm{Path coefficients}:P_{ij}=r_{ij}P_{j}$$

(3) $$\;\mathrm{Determination coefficients of single factors to}\;Y:d_{i}=P_{i}^{2}$$

(4) $$\mathrm{Determination coefficients of}\;\mathrm{two}\;\mathrm{factors to}\;Y:d_{\mathrm{ij}}=2r_{\mathrm{ij}}P_{i}P_{j}$$

(5) $$\mathrm{The residual path coefficient}:P_{e}=\sqrt{1-\sum d}$$

Annotation:

*r*_ij_: the correlation coefficients between all factors.

*P*_i_: the influence degree of single factor acting independently on *Y*.

*P*_j_: the influence degree of other factors acting independently on *Y* (*i* ≠ *j*).

*P*_ij_: the influence of one factor *X*_i_ on *Y* through another factor X_j_ (*i* ≠ *j*).

*d*_i_: the influence of single factors on *Y*.

*d*_ij_: the influence of two factors on *Y*.

*P*_e_: the coefficients of residual factors that not consider during the process of analysis.

## Results and discussion

### Changes in activity of enzymes related to browning and pH value during fermentation

**Figure 1:**
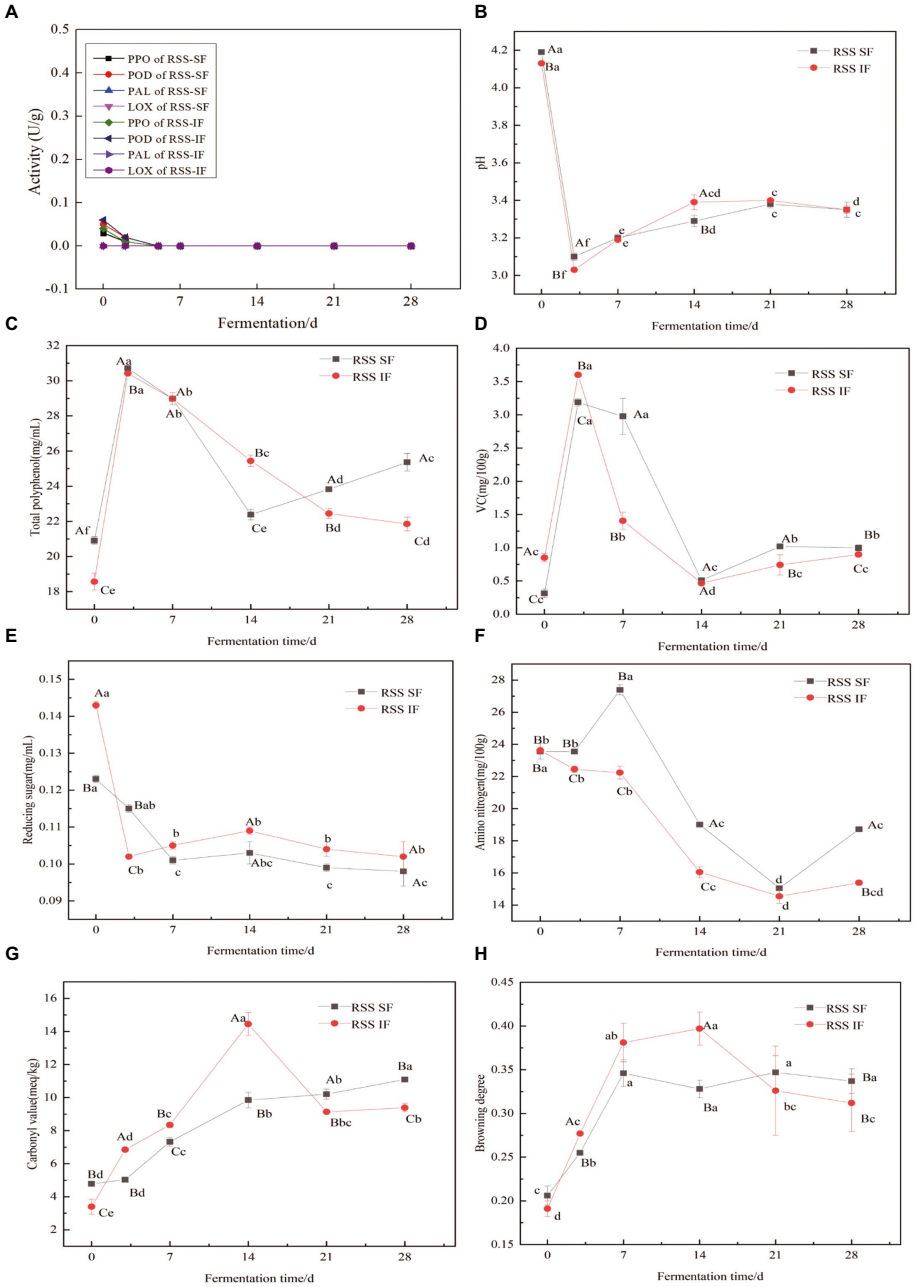
Changes of, PPO, POD, PAL, and LOX activity **(A)**, pH **(B)**, total polyphenol **(C)**, Vc **(D)**, reducing sugar **(E)**, amino nitrogen **(F)**, and carbonyl value **(G)** browning degree **(H)** of group RSS-SF (red sour soup with spontaneous fermentation) and RSS-IF (red sour soup with inoculant fermentation) during fermentation. Different capital letters represent significant differences at the same time and different groups, lower case letters indicate the difference is significant at same group and different fermentation time of *p* < 0.05.

Generally, browning of food was mainly composed of enzymatic and non-enzymatic browning. For enzymatic browning, those enzymes, such as PPO, POD, and PAL, were considered to be the most important factors associated with browning (19). PPO shows either mono-or di-phenol oxidase activity that could catalyze oxidation of phenolic compounds and convert into pigments (19). Besides, it is reported that POD exhibits synergistic action with PPO during the process of browning. Different from PPO and POD, PAL is a committed enzyme in phenyl propanoid metabolism and its activity is related to the concentration of phenolic compounds which are the substrates for PPO and POD (19). Previous studies indicated that the activities of PPO, POD, and PAL are closely related with the browning of different vegetables and fruits (20). In order to investigate the relationship of PPO, POD, and PAL with browning of RSS, the activities of those enzymes were determined during the process of fermentation. As shown in Figure 1A, no enzyme activities of PPO, POD, and PAL were detected during the process of whole fermentation. Research has shown that PPO, POD, and PAL are easily deactivation at high temperature (above 60°C) and acid conditions (below pH4.0) (19, 21). In this study, the final step of preparation of RSS was incubated at 85°C for 20 min that may denature completely of PPO, POD, and PAL. Meanwhile, the values of pH were totally below 4.0 during the process of fermentation (Figure 1B). The activities of PPO, POD, and PAL indicated that browning of group RSS-SF and RSS-IF may not related to enzymatic browning.

### Changes in total polyphenol and ascorbic acid during fermentation

The results of changes in total polyphenol during fermentation were depicted in Figure 1C. Interestingly, at the first stage of fermentation (from 0 to 3 days), the contents of total polyphenol from group RSS-SF and RSS-IF were gradually increased, which is similar to previous research (22). As the fermentation time above 3 days, the concentration of total polyphenol was gradually decreased and kept in the ranges from 21.38 to 28.98 mg/mL. It is well known that polyphenol is a critical factor related to browning of different food (20). The polyphenol are easily oxidized and further polymerized to form macromolecular brown compounds, which cause color changes. Generally, the oxidation of polyphenols can be divided into enzymatic and non-enzymatic oxidation of polyphenols (23). In this study, oxidation of polyphenol was mainly caused by non-enzymatic. On the one hand, degradation of polyphenol could react at a slow speed in acidic conditions due to its multiple weakly acidic phenolic hydroxyl groups (24). On the other hand, the multiple phenolic hydroxyl groups had strong reducibility which could cause the polyphenol automatic oxidation and decrease the content (25).

As natural antioxidant, Vc is one of the main nutrition components of different food and also closely related to non-enzymatic browning during the process of food production. According to the previous research, the degradation of Vc could convert to derivative and intermediate to react with other components to lead to browning (26). In summary, there are two pathways of Vc oxidation, which include the aerobic and anaerobic degradation pathways. Therefore, determination of changes in Vc is helpful to elucidate the browning of RSS. As depicted in Figure 1D, the changes of Vc were similar to total polyphenol during the whole process of fermentation. The concentrations of Vc of group RSS-SF and RSS-IF were gradually increased at the initial stage of fermentation (from 0 to 3 days). Then, the concentration of Vc of group RSS-SF and RSS-IF gradually decreased and kept in the ranges from 0.85 to 2.97 mg/100 g when the fermentation time above 3 days.

### Changes in reducing sugars and amino nitrogen during fermentation

Previous studies demonstrated that Maillard reaction is closely associated with non-enzymatic browning during the storage of many foods (9, 27). As the most important precursors of Maillard reaction, amino acids and reducing sugars could undergo browning reaction to influence the color of different foods. The changes in in reducing sugars and amino nitrogen during fermentation were illustrated in Figures 1E, F. The content of reducing sugar in both groups were keeping decreasing during whole fermentation time and shown obviously difference at the day of 0 and 14 days (*p* < 0.05). Additionally, the amino nitrogen content in group RSS-SF increased after 7 days, and then decreased until 21 days, while the group RSS-IF continually decreased until 21 days, and with a slight increase at 28 days (Figure 1F).

### Changes in carbonyl value during fermentation

Generally, the carbonyl value is related to the oxidation of lipid. Previous study demonstrated that oxidation of lipid is involved with browning of food (11). In summary, the whole networks of lipid oxidation to final colored products include different reactions which are mainly divided into three steps: (i) oxidation of lipid to form lipid hydroperoxides; (ii) rearrangement, fragmentation or degradation of lipid hydroperoxides to produce variety of volatiles and non-volatile monomers; (iii) aldol condensation and/or carbonyl-amine polymerization of products from second step to form colored polymers (11). Briefly, lipids can be oxidized by two main ways which include non-enzymatic oxidation and enzymatic-catalyzed oxidation (11). In this study, the oxidation of lipid is mainly non-enzymatic autoxidation due to no enzyme activities of LOX were detected during the whole fermentation (Figure 1A). As depicted in Figure 1G, the carbonyl value in group RSS-SF increased continually during the whole process of fermentation. Different from group RSS-SF, the carbonyl value of group RSS-IF reached maximum (14.4 meq/kg) at 14 days and then decreased to 9.4 meq/kg as the fermentation time reached 28 days. It is reported that oxidation of lipid is involved with non-enzymatic browning (11). The lipid oxidation occurred during fermentation may contribute to the non-enzymatic browning of RSS-SF and RSS-IF.

### Browning degree change during fermentation

The changes in browning degree of different sample were shown in Figure 1H. For group RSS-SF, the value of browning degree increased gradually from 0 to 7 days. The values of browning degree at 0 and 7 days were 0.206 and 0.346, respectively. As the fermentation time above 7 days, the value of browning degree changed little and kept in the range from 0.328 to 0.346 (Figure 1H). Similar to group RSS-SF, the value of browning degree of group RSS-IF also increased gradually from 0 to 14 days. For group RSS-IF, the highest value of browning degree was 0.397 when the fermentation time was 14 days (Figure 1H). However, as the fermentation time above 14 days, the value of browning degree decreased slowly and reached 0.312 at the end of fermentation (Figure 1H).

Based the results of activities of PPO, POD, and PAL (Figure 1A), we conclude that browning of group RSS-SF and RSS-IF during fermentation is non-enzymatic browning. Generally, non-enzymatic browning is composed of oxidation of polyphenol, degradation of ascorbic acid, Maillard reaction and lipid oxidation (10). According to the obtained results, we deduced that the main factors related to non-enzymatic browning of group RSS-SF and RSS-IF maybe oxidation of polyphenol, degradation of ascorbic acid and lipid oxidation. However, it is essential to figure out exactly the main reasons for non-enzymatic browning of group RSS-SF and RSS-IF by a statistical method.

### Path analysis among various factors related to non-enzymatic browning

Previous research indicated that path analysis is a suitable method for analysis and isolation of the crucial factors closely associated with browning (10). Usually, the process of path analysis includes analysis and calculation of correlation coefficients, direct coefficients and determination coefficients. The value of determination coefficients exhibits the contribution of different factors related to the final results. In this study, the correlation coefficients of different factors related to browning degree were firstly analyzed and the results shown in Table 1. Based on the results of correlation coefficients (Table 1), the direct path coefficients were secondly calculated. As shown in Table 2, the order of direct path coefficients of group RSS-SF was *P*_4_ > *P*_5_ > *P*_3_ > *P*_2_ > *P*_1_, which exhibited the main direct browning factor was degradation of Vc, followed by oxidation of lipid and polyphenol. For group RSS-IF, the order of direct path coefficients was *P*_4_ > *P*_3_ > *P*_2_ > *P*_5_ > *P*_1_, which indicated that degradation of Vc and oxidation of polyphenol played an important role in browning.

**Table 1:** Correlation coefficients in group RSS-SF and RSS-IF.

Different group	Factors	*X*_1_	*X*_2_	*X*_3_	*X*_4_	*X*_5_	*Y*
RSS-SF	*X*_1_	1	0.407	0.474^*^	0.597^**^	−0.720^**^	−0.362
	*X*_2_	0.407	1	−0.105	−0.030	−0.793^**^	−0.869^**^
	*X*_3_	0.474^*^	−0.105	1	0.966^**^	−0.239	0.160
	*X*_4_	0.597^**^	−0.030	0.966^**^	1	−0.376	0.100
	*X*_5_	−0.720^**^	−0.793^**^	−0.239	−0.376	1	0.807^**^
RSS-IF	*X*_1_	1	0.493^*^	0.247	0.527^*^	−0.703^**^	−0.464
	*X*_2_	0.493^*^	1	−0.629^**^	−0.281	−0.587^*^	−0.675^**^
	*X*_3_	0.247	−0.629^**^	1	0.688^**^	0.261	0.488^*^
	*X*_4_	0.527^*^	−0.281	0.688^**^	1	−0.339	−0.225
	*X*_5_	−0.703^**^	−0.587^*^	0.261	−0.339	1	0.806^**^

RSS-SF, red sour soup with spontaneous fermentation; RSS-IF, red sour soup with inoculant fermentation. *X*_1_, Amino nitrogen; *X*_2_, Reducing sugar; *X*_3_, Total polyphenol; *X*_4_, Vc, *X*_5_: Carbonyl value; *Y*, Browning degree. ^**^extremely Significant correlation (*p* < 0.01). ^*^Significant correlation (*p* < 0.05).

**Table 2:** Path analysis in group RSS-SF and RSS-IF.

Different group	Factors	*X*_1_	*X*_2_	*X*_3_	*X*_4_	*X*_5_
RSS-SF	*X*_1_	0.079^*^	−0.048	−0.500	0.878	−0.771
	*X*_2_	0.032	−0.119^*^	0.111	−0.044	−0.849
	*X*_3_	0.038	0.012	−1.054^*^	1.420	−0.256
	*X*_4_	0.047	0.004	−1.019	1.470^*^	−0.403
	*X*_5_	−0.057	0.094	0.252	−0.553	1.071^*^
RSS-IF	*X*_1_	0.113^*^	−0.200	0.190	−0.463	−0.104
	*X*_2_	0.056	−0.406^*^	−0.485	0.247	−0.087
	*X*_3_	0.028	0.255	0.770^*^	−0.604	0.039
	*X*_4_	0.060	0.114	0.530	−0.878^*^	−0.050
	*X*_5_	−0.079	0.238	0.201	0.298	0.148^*^

RSS-SF, red sour soup with spontaneous fermentation; RSS-IF, red sour soup with inoculant fermentation. *X*_1_, Amino nitrogen; *X*_2_, Reducing sugar; *X*_3_, Total polyphenol; *X*_4_, Vc, *X*_5_: Carbonyl value; *Y*, Browning degree. The number with ^*^ is direct path coefficients (P_i_). The rest number is indirect path coefficients (*P*_ij_).

The determination coefficients of different factors related to browning were calculated based on the results of direct path coefficients (Table 2) through the formulas from (2) to (4) and the results were shown in Table 3. The top three of determination coefficients in group RSS-SF were *d*_34_ > *d*_4_ > *d*_45_, which suggested that the main browning factors were the interaction between oxidation of polyphenol and degradation of Vc, then came after the degradation of Vc. Meanwhile, the top three of determination coefficients in group RSS-IF were *d*_34_ > *d*_4_ > *d*_3_, which indicated that the main browning factors also included the interaction between oxidation of polyphenol and degradation of Vc, degradation of Vc, and oxidation of polyphenol. In summary, non-enzymatic browning is complicated and always involved with multiple reactions such as Maillard reaction, degradation of Vc, oxidation of polyphenol and lipids (10, 23). In this study, the main factors related to non-enzymatic browning of group RSS-SF and RSS-IF during fermentation were oxidation of polyphenol and degradation of Vc.

**Table 3:** Determination coefficients in group RSS-SF and RSS-IF.

Determination coefficients	RSS-SF	RSS-IF
*d*_1_	0.006	0.013
*d*_2_	0.014	0.165
*d*_3_	1.112	0.593
*d*_4_	2.161	0.771
*d*_5_	1.146	0.022
*d*_12_	−0.008	−0.045
*d*_13_	−0.079	0.043
*d*_14_	0.139	−0.105
*d*_15_	−0.122	−0.024
*d*_23_	−0.026	0.393
*d*_24_	0.010	−0.200
*d*_25_	0.202	0.071
*d*_34_	−2.995	−0.931
*d*_35_	0.540	0.060
*d*_45_	−1.184	0.088
∑d	0.917	0.915
*P_e_*	0.288	0.292

RSS-SF, red sour soup with spontaneous fermentation; RSS-IF, red sour soup with inoculant fermentation. *d*_1_ to *d*_5_ represent the determination coefficient of amino nitrogen, reducing sugar, total polyphenol, Vc and carbonyl value to browning degree (*Y*), respectively. *d*_12_ to *d*_15_ represent the interaction between amino nitrogen and reducing sugar, total polyphenol, Vc and carbonyl value to browning degree (*Y*), respectively. *d*_23_ to *d*_25_ represent the interaction between reducing sugar and total polyphenol, Vc and carbonyl value to browning degree (*Y*), respectively. *d*_34_ to *d*_35_ represent the interaction between total polyphenol and Vc and carbonyl value to browning degree (*Y*), respectively. *d*_45_: the interaction between Vc and carbonyl value to browning degree (*Y*).

## Conclusion

In conclusion, no enzyme activities of PPO, POD, and PAL were detected during the processes of whole fermentation revealed that browning of group RSS-SF and RSS-IF were mainly non-enzymatic browning. Moreover, path analysis was used to analyze the main factors related non-enzymatic browning of group RSS-SF and RSS-IF. The main browning factors of group RSS-SF and RSS-IF were composed of oxidation of polyphenol and degradation of Vc. The results of this study will provide a foundation for further control of browning of RSS during fermentation.

## Data availability statement

The original contributions presented in the study are included in the article/Supplementary material, further inquiries can be directed to the corresponding authors.

## Author contributions

ZL: writing–original draft, methodology, conceptualization, visualization, and data curation. XZ: methodology. MW, ZG, and BL: writing–review and editing. LZ: writing–review and editing and resources. JW: writing–original draft, conceptualization, validation, and supervision. All authors contributed to the article and approved the submitted version.

## Funding

This work was supported by the science and technology innovation program of Hunan Province (2019TP1028, 2019SK2122, 2019NK4229, and 2022NK2039) and Postgraduate Scientific Research Innovation Project of Hunan Province (CX2021SY064).

## Conflict of interest

Author JW was employed by Shenzhen Raink Ecology & Environment Co., Ltd.

The remaining authors declare that the research was conducted in the absence of any commercial or financial relationships that could be construed as a potential conflict of interest.

## Publisher’s note

All claims expressed in this article are solely those of the authors and do not necessarily represent those of their affiliated organizations, or those of the publisher, the editors and the reviewers. Any product that may be evaluated in this article, or claim that may be made by its manufacturer, is not guaranteed or endorsed by the publisher.

## Supplementary material

The Supplementary material for this article can be found online at: https://www.frontiersin.org/articles/10.3389/fnut.2023.1092745/full#supplementary-material
