# Supplementary material for: Analysis of factors related to browning of red sour soup during fermentation
Source: Front Nutr. 2023 Feb 28;10:1092745. doi: 10.3389/fnut.2023.1092745 (PMC10012660; doi:10.3389/fnut.2023.1092745)
Supplement: Supplementary file 1 [file Table_1.DOCX]

1. **Determination of activity of enzymes (PPO, POD and PAL)**

PPO activity was determined according to a previously described method with slight modifications (Palamutoğlu R, 2020). 10.0 g red sour soup was homogenized in 20 mL of 0.2 mol /L phosphate buffer (pH 6.8). After centrifuging (Velocity 14R, Dynama Australia, Australia) at 10,000 × g for 10 min at 4 ℃, the supernatant was collected for PPO activity determination. A 3 mL reaction mixtures included 0.20 mL of the supernatant,1.8 mL phosphate buffer (pH 6.8) and 1.0 mL of 0.2 mol/L oxophenic acid solution. Immediately, the changes in absorbance at 410 nm was recorded within 3 minutes.

$X(U/g)=\frac{\Delta A}{0.001\times t\times m}$ ⑴

Where $\Delta A$ represents the absorbance difference within 3 minutes; t was the reaction time; m is the mass of sample.

Peroxidase (POD) activity was determined by the method described as (Terefe, Tepper, Ullman, Knoerzer and Juliano, 2016) with slight modifications. The sample pretreatment was the same as the PPO activity. A 3.5 mL reaction mixtures included 1 mL phosphate buffer (pH 6.8), 0.5 mL of 0.10 mol/L guaiacol solution, 1 mL of 0.2 mol/L hydrogen peroxide solution and 1 mL of the supernatant. An increase in absorbance at 436 nm wavelength within 3 minutes was recorded.

$X(U/g)=\frac{\Delta A}{0.001\times t\times m}$ ⑵

Where $\Delta A$ represents the absorbance difference within 3 minutes; t was the reaction time; m is the mass of sample.

Phenylalanine ammonia lyase (PAL) activity was determined according to the method of (Liu et al., 2018) with some modifications. 10.0 g red sour soup was homogenized in 10.0 mL boric acid buffer (pH8.8). After centrifuging (Velocity 14R, Dynama Australia, Australia) at 10,000 × g for 10 min at 4 ℃, the supernatant was collected to evaluate the PAL activity. A 3.6 mL reaction mixtures contained 0.8 mL of the supernatant, 2 mL of 1 mol/L boric acid buffer (pH 8.8) and 0.8 mL of 0.20mol/L L-phenylalanine solution. The mixture was blended and incubated at 40℃ for 60 min. The PAL activity was determined at a wavelength of 290 nm.

1. **Determination of amino nitrogen, carbonyl value and Vc**

Amino nitrogen was recorded according to GB/T 5009.235-2016. 10.0 g red sour soup was mixed with 20mL deonized water and titrated to 8.2 with 1.0mol/L sodium hydroxide solution,the volume of consumed NaOH was recorded as V_1_.then added 10mL neutral formaldehyde into it and continually titrated with 1.0mol/L sodium hydroxide solution until the pH meter (FiveEasy Plus FE28, Mettler Toledo Instruments Co., Ltd., Shanghai, China) revealed 9.2.The the volume of consumed NaOH was recorded as V_2_.

$X(mg/100g)=C\times(V_{2}-V_{1})\times100\times0.014\times F$ ⑶

where C was the concentration of sodium hydroxide solution and F was the dilution multiple of sample.

Carbonyl value was analyzed by the method described as GB 5009.230-2016 (National Health and Family Planning Commission of the People’s Republic of China, 2016c). 40.0 g red sour soup was transferred into conical flask containing 20 mL petroleum benzin and stewed 18 hours. Samples were filtered and put into thermostat water bath (DZKW-4, Beijing Zhongxing Weiye Instrument Co., Ltd., Beijing, China) at 90 ℃ to remove the residual petroleum benzin. 0.50 g oil sample was weighed into a 25mL tube containing 5 mL petroleum benzin that was used to dissolve the oil. Then 3 mL trichloroacetic acid solution and 5 mL 2,4-dinitrophenylhydrazine solution were added to the mixture and placed in a 60 ℃ water bath for 30min and then cooled to room temperature. 10 mL potassium hydrate-alcohol solution was slowly added to the mixture. The absorbance of each sample was measured at a wavelength of 440 nm with the blank test that was only adding corresponding chemicals at the same time. The results were calculated as

$x(meq/kg)=\frac{A}{854\times m}\times1000$ ⑷

where A is the absorbance at 440 nm, 854 is the average absorbance of various aldehydes, m is mass of sample,1000 is conversion coefficient.

Vc content was measured using the method described by GB 5009.86-2016 (National Health and Family Planning Commission of the People’s Republic of China, 2016b). 10.0 g red sour soup was weighed into a beaker and transferred into 100 mL volumetric flask with oxalic acid solution. Shaking it well and filter. Then 10 mL filtrate was accurately sucked into 50 mL conical flask and titrated with calibrated 2,6-dichloroindophenol solution until the solution turned into pink and kept for 15s. At the same time the blank(V_0_) test was conducted . The following formula was used to calculate the content of Vc:

$X(mg/100g)=\frac{(V-V_{0})\times T\times A}{m}\times100$ ⑸

where V is 2,6-dichloroindophenol solution volume of samples consumed (mL); T is the titer of 2,6-dichloroindophenol solution; A is dilution ratio of sample; m is mass of sample.

1. **Determination of reducing sugar and total polyphenol**

Reducing sugar was determined according to the method described previous (Yang et al., 2017) with slight modifications. 1 mL of the supernatant was transferred into 4 mL of DNS reagent tube and then the mixtures were heated in boil water for 5 minutes. After cooling down to room temperature and diluting with deionized water to 10 mL, the absorbance was recorded at a wavelength of 540nm.

10.0 g red sour soup was homogenized in 20mL of 50% alcohol and kept at 180 rpm for 20 min and then the mixtures were centrifuged (Velocity 14R, Dynama Australia, Australia)) at 10,000 × g at 4 ℃ for 10 min. The supernatant was determined at a wavelength of 765 nm using (Derakhshan et al., 2018).
